# Ugi and Groebke–Blackburn–Bienaymé Reactions in Medicinal Chemistry: An Outlook on New Bioactive Compounds

**DOI:** 10.1021/acsomega.5c06428

**Published:** 2025-10-09

**Authors:** Gustavo Barbosa-Reis, Julia Lavorenti, Bruna Lorentz Alves, Diogo Oliveira-Silva

**Affiliations:** Department of Chemistry - Institute of Environmental, Chemical and Pharmaceutical Sciences, 28105 Federal University of São Paulo (UNIFESP), Diadema Campus, 275 Artur Riedel St., Diadema 09972-270, Brazil

## Abstract

The FDA has approved 174 new molecular entities over the last 5 years. In 2023, the anticancer and infectious disease areas represented an impressive percentage of drug approvals, 24% and 9%, respectively, following the decade’s trends. This reflects the pharmaceutical industry’s increasing demand for novel compounds, particularly in these fields. Isocyanide-based multicomponent reactions, such as Ugi and Groebke–Blackburn–Bienaymé reactions, stand out for their versatility in obtaining different bioactive scaffolds using simpler starting materials. A large array of complex scaffold molecules can be obtained by using a multicomponent synthesis step. For this reason, this mini-review summarizes some recent works that employed this approach to afford new anticancer, antiviral, antimicrobial, and antiparasitic compounds. In each case, how the modifications impact activity or cytotoxicity is discussed. Also, the possible gaps that should be evaluated in further work are highlighted to inspire the design of new compounds.

## Graphical abstract

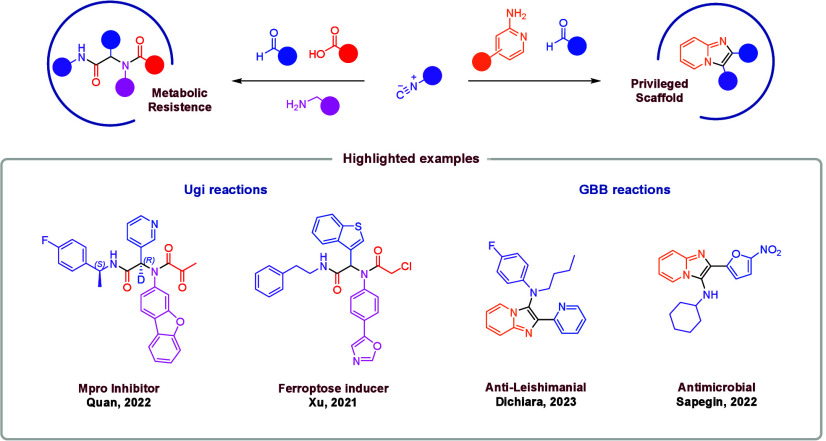

## 1 Introduction

Multicomponent reactions (MCRs) are synthetic processes in which three or more starting materials react in a one-pot reaction step to afford a single product. This approach is becoming relevant due to the atom economy and the ability to access complex scaffolds from simple starting materials. The use of multicomponent reactions started with the Strecker reaction in 1850. Since then, other variants of MCRs have been reported.^1^ Isocyanide-based multicomponent reactions include Passerini, Ugi four-component and its variations like Ugi–Tetrazol and Ugi–Smiles, and Groebke–Blackburn–Bienaymé (GBB). In the last 25 years, more than 3946 publications about these five isocyanide-based multicomponent reactions were published and indexed in the Web of Science. The keywords and number of results obtained in bibliography research in Web of Science were “Passerini reaction” (960 publications), “Ugi reaction” (2621 publications), “Ugi tetrazoles reaction” (129 publications), “Groebke Blackburn Bienaymé reaction” (158 publications), and “Ugi smiles reaction” (78 publications). These numbers indicate the high interest of the scientific community in multicomponent reactions. However, Passerini reactions lead to α-acyloxy amide derivatives that present an ester group that is metabolically unstable, which could be relevant in a prodrug design context. In this work, the use of Ugi and GBB reactions in medicinal chemistry will be highlighted because they can be used as powerful tools to obtain new biological compounds by the relevant scaffold characteristics and by their metabolic stability.

The Groebke–Blackburn–Bienaymé three-component reaction (GBB-3CR), published nearly simultaneously by Groebke, Blackburn, and Bienaymé, uses amidines, aldehydes, and isocyanides as starting materials to obtain imidazo­[1,2-*a*]-pyridine derivatives. The reaction mechanism (Figure 1) begins with the formation of the imine (**A**), resulting from condensation of the aldehyde and the amine. The imine is activated (**B**) by a Lewis or Brønsted–Lowry acid, and the isocyanide leads to the [4 + 1] cycloaddition followed by aromatization via a 1,3-H shift, yielding the final molecule (**C**).^2^ Due to the variety of possible starting materials, this method enables the acquisition of a large range of compounds. This scaffold is considered privileged in medicinal chemistry because the pyridinic core is a well-known pharmacophore with multiple therapeutic targets, showing a high biological activity and structural diversity.^3,4^

**Figure 1:**
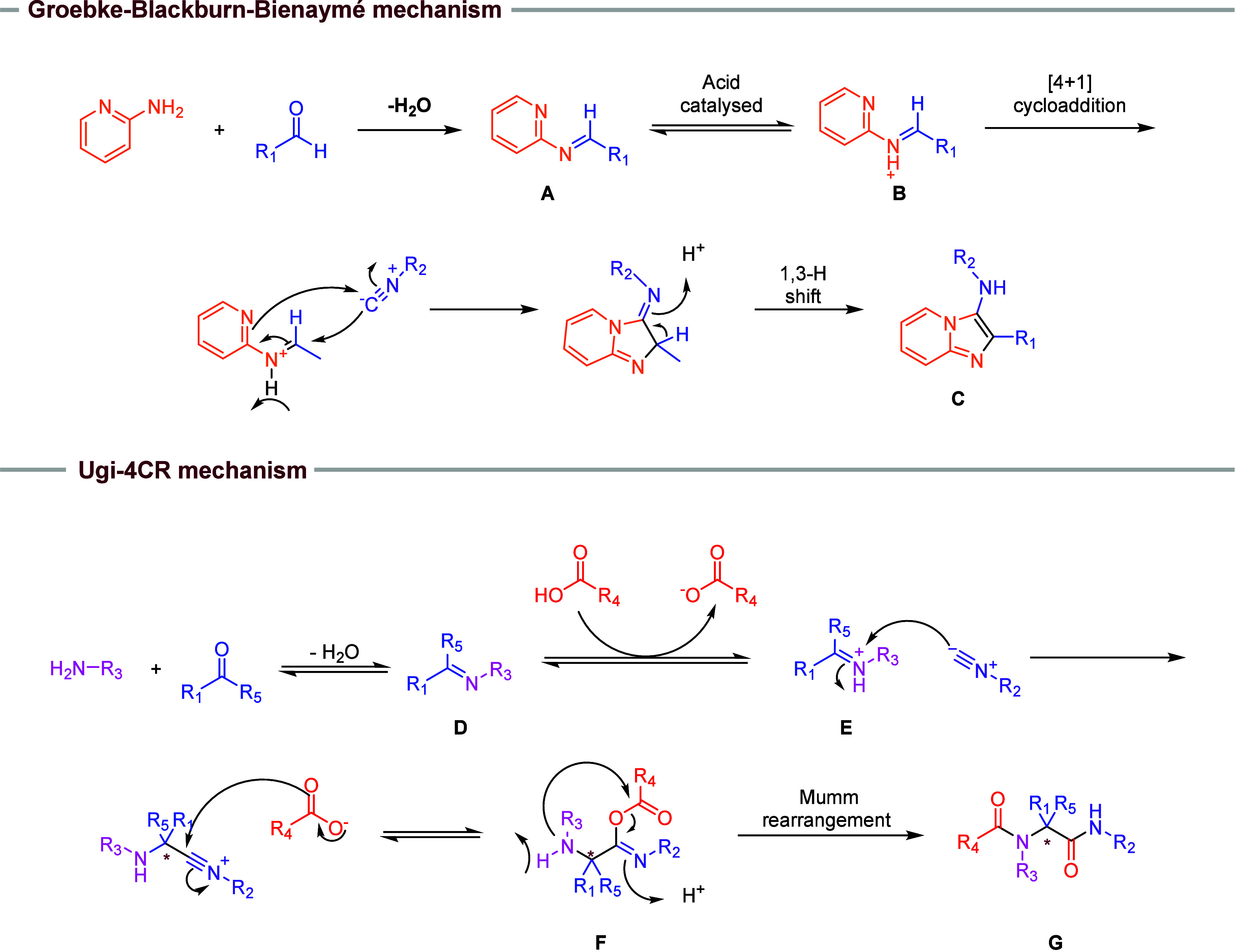
Mechanism of GBB and Ugi four-component reactions.

The Ugi four-component reaction uses primary amines, oxo compounds (aldehydes or ketones), carboxylic acids, and isocyanides to obtain peptoids (or pseudopeptides) as products. The reaction mechanism (Figure 1) begins with the formation of the imine (**D**) from the condensation of aldehyde with amine, and protonation by carboxylic acid results in the iminium ion (**E**). This ion undergoes an irreversible nucleophilic attack by isocyanide and subsequently by the carboxylate ion to generate the intermediate α-adduct (**F**). Finally, a Mumm rearrangement is responsible for the formation of the peptoid (**G**).^5^ Due to their distinct nitrogen substitution patterns, peptoids exhibit improved resistance to proteolysis compared to peptides, an important trait for biological activities. The robustness of the Ugi-4CR, along with its variations (e.g., postcondensations, Ugi-Azide, Ugi-Smiles, etc.), makes it convenient for accessing a broad spectrum of scaffolds, which is attractive to medicinal chemistry for optimizing synthetic routes and discovering new biologically active compounds.^6^

A drug is a foreign molecule that impacts biological processes which are used to treat or prevent disease.^7^ These compounds can be synthesized or can be a natural product. Among the main goals, the optimal drug needs to be chemically and metabolically stable, should have a specific action and be safe and nontoxic.^7^ In many cases, new generations of drugs for many diseases are developed, aiming to increase the activity, decrease the cytotoxicity, or both at the same time. In bacterial infections, for example, the design of new compounds is important due to the antimicrobial resistance of some microorganisms. Penicillin was the first compound with antimicrobial activity reported in 1928. Almost 100 years later, recent research showed that 75% of infections by *Enterococcus faecium*, a multidrug-resistant bacteria and hospital-acquired pathogen, are from penicillin-resistant strains.^8^ If there were no other antimicrobial compounds available in the market, this type of infection would lead to a higher number of deaths worldwide.

In 2023, the FDA approved 53 new drugs. The oncology field approved the largest number of new drugs (13 drugs, 24%), followed by neurology (9 drugs, 16%) and infectious disease (5 drugs, 9%).^9^ This interest is also reflected in the number of publications. A bibliographic search on Web of Science, conducted in this mini-review, found up to 181,000 publications in oncology and infectious disease (anticancer, antiviral, antimicrobial, and antiparasitic) during a five-year period (Jan-2020 to Jan-2025), corroborating the trends in FDA drug approvals (Figure 2).

**Figure 2:**
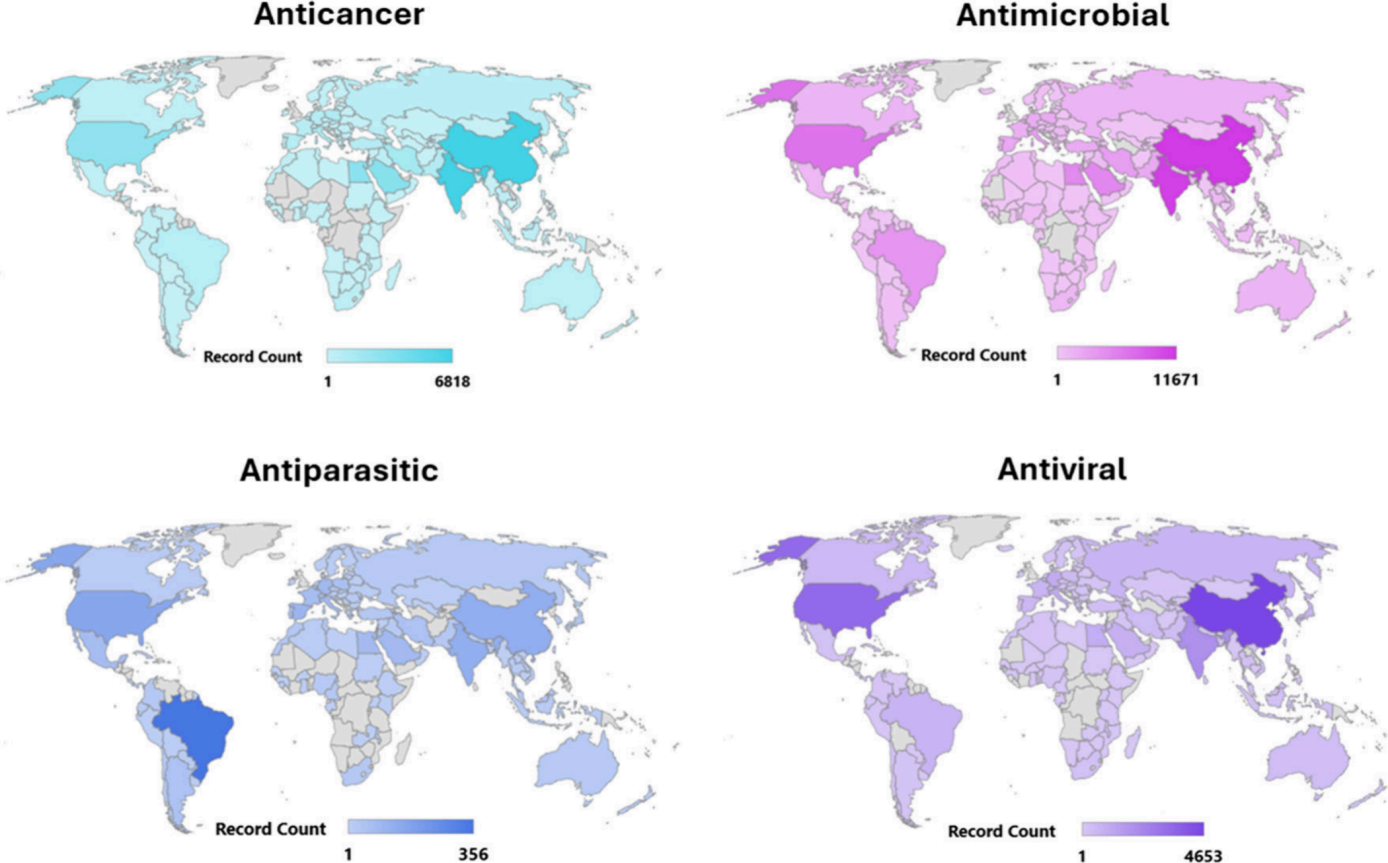
World map distribution of publications in oncology and infectious disease. In the last 5 years, up to 181,000 publications were reported. Antimicrobial has the highest number of publications (57.41%), followed by anticancer (26.33%), antiviral (14.86%), and antiparasitic (1.38%). The world maps were plotted based on publications indexed in research in the Web of Science platform since 1900, using “anticancer activity”, “antimicrobial activity”, “antiviral activity”, and “antiparasitic activity” as keywords.

This mini-review is our contribution to discussing the applications of Ugi and GBB reactions in medicinal chemistry, with a focus on treatments for infectious diseases (microbial, viral, and parasitic) and cancer. Herein, new studies are compiled and suggestions for further research are presented. This emphasis is justified by the numerous opportunities in oncology and the urgent need for new drugs to address neglected diseases such as parasitic infections.

## 2 Infectious Diseases

Infectious diseases are caused by organisms, such as bacteria, fungi, viruses, or parasites. These diseases can be transmitted through person-to-person contact, insect or animal vectors, consumption of contaminated food and water, or environmental exposure. Despite the availability of vaccines and drugs to control these infections, they remain a critical concern in countries with low to medium mortality rates. The ongoing search for new drugs is crucial to controlling and combating these diseases.^10^

### 2.1 Parasitic Infections

#### 2.1.1 Leishmaniasis

Leishmaniasis is a neglected tropical disease that affects tropical countries and can manifest in three forms: cutaneous, mucocutaneous, and visceral. Between 2015 and 2019, one billion people worldwide have received chemotherapy for this disease. However, the current antileishmaniasis drugs are outdated and often present severe side effects or resistance issues in some species. Therefore, the development of new antileishmaniasis compounds for alternative treatment is tremendously relevant.^11–13^

Akao and co-workers^11^ used the GBB methodology to obtain several imidazo­[1,2-*a*]­pyridine derivatives with antileishmaniasis (*L. donovani*) activity. Among these, 2-pyridinecarboxaldehyde derivatives showed the most relevant activities (Figure 3). Chlorine and methoxy substituents were tolerated (compounds **1**–**4**). However, changing the nitrogen position (3 or 4-pyridinecarboxaldehyde derivatives) resulted in a loss of activity. Replacing the pyridyl ring with a 5-membered ring often led to a slight or complete loss of activity, as seen in compound **6**. Additionally, the bioisosteric replacement of the pyridyl ring with phenyl rings containing electron-donating groups (EDGs) showed moderate activity and favorable cytotoxicity values. Moreover, the antileishmaniasis characteristics are unlikely to be correlated with the bidentate metal chelation effect, as exemplified by compound **5**, which displayed higher potency when compared with **6**. The influence of more lipophilic and heterobiaryl aldehydes should be evaluated in new studies. Also, the presence of EDG groups in imidazo­[1,2-*a*]­pyridines increases the activity, while electron-withdrawing groups (EWGs) decrease. However, only positions 2 and 3 in the ring and a few EDG/EWG substituents were evaluated, lacking examples on other positions and examples of other amidines to determine the influence in the activity. In isocyanide influence, it was observed that the use of aliphatic cycloalkanes increases activities while aliphatic heterocycles decrease. The replacement of aryl isocyanides leads to the most active compounds, and the presence of EDG and bulky groups were tolerated. However, more examples of EDG/EWG, bulky groups, and *N*-alkylation processes (aiming to increase the lipophilic characteristic) should be considered in new studies to verify the influence in activity and cytotoxicity.

**Figure 3:**
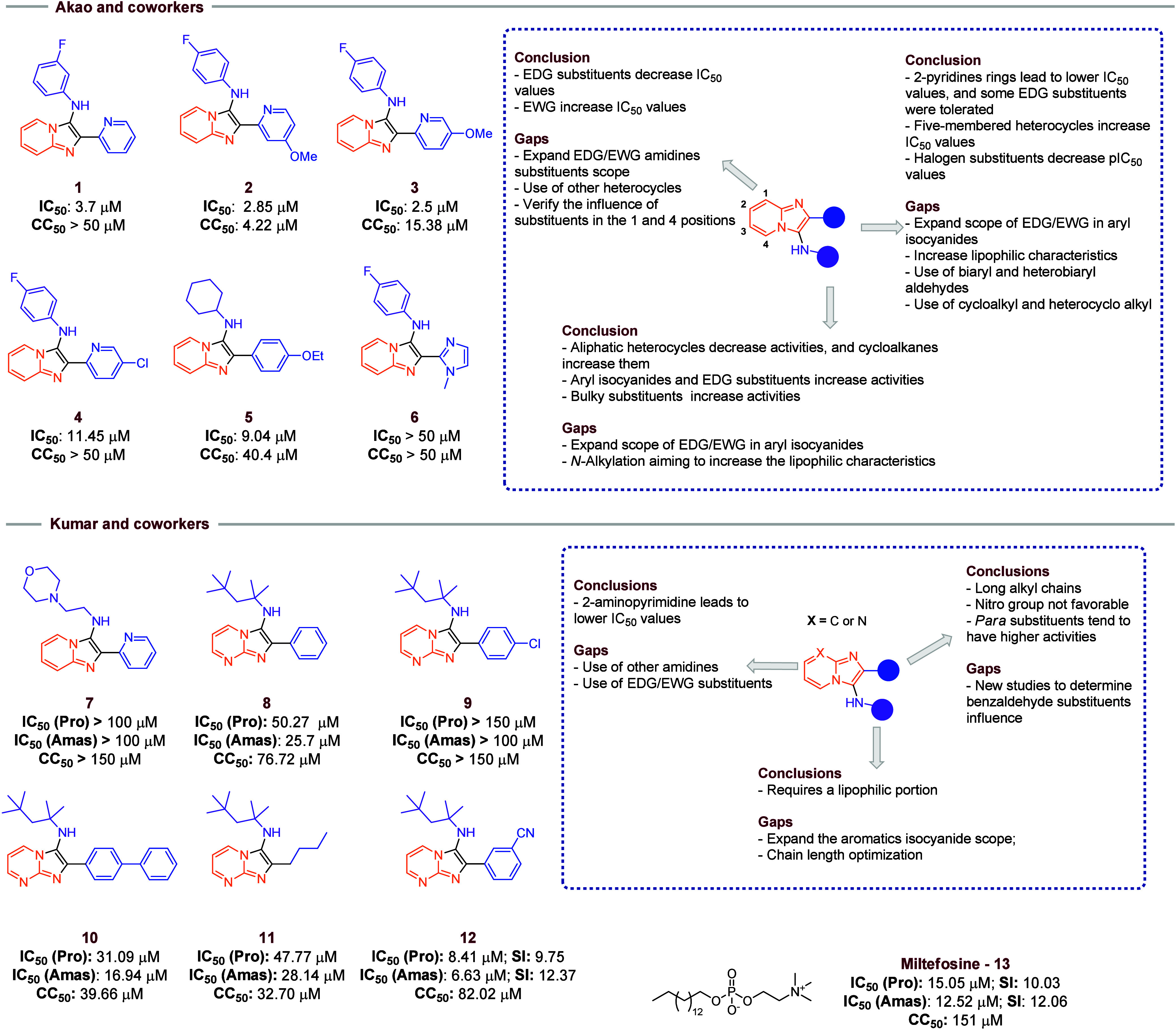
Selected compounds of Akao and co-workers and Kumar and co-workers. In both sections, the principal’s compounds’ characteristics are highlighted and how they affect the IC_50_ and CC_50_ values. Also, there is a summary of possibilities of some modifications to be considered in new studies. IC_50_ = half maximal inhibitory concentration. CC_50_ = 50% cytotoxic concentration. EDG = electron-donating groups. EWG = electron-withdrawing group.

Kumar and co-workers^13^ (Figure 3) employed the GBB reaction to synthesize a set of imidazole­[1,2-*a*]­pyridine derivatives with a morpholine in the isocyanide moiety (compound **7**), due to its presence in antiparasitic compounds, and evaluated the profile against *L. amazonensis*. Eight compounds with this isocyanide were synthesized, but no antileishmaniasis activity was found in either promastigote or amastigote forms. However, the morpholine replacement by a more hydrophobic group, such as 1,1,3,3-tetramethylbutyl isocyanide (compound **8**), resulted in compounds with moderate activity against the promastigote form. The increase of lipophilic characteristics using other alkyl isocyanides and the evaluation of aryl isocyanides need to be considered in further work. Furthermore, testing other aldehydes (compounds **9**–**12**) revealed that 3-cyanobenzaldehyde afforded the product with the best activity (compound **12**), with IC_50_ (Pro): 8.41 μM and IC_50_ (Amas): 6.63 μM, which showed better IC_50_ values and similar selectivity index (SI) in comparison to Miltefosine (**13**), a drug used in leishmaniasis treatment. *Para*-substituents in aldehydes lead to the most active compounds; however, new studies with other EDGs and EWGs must be considered for further evaluations. Also, the use of 2-aminopyrimidines leads to a decrease in the IC_50_ values, which indicates that modifications in these portions can be studied.

Ceravolo and co-workers^14^ used the Ugi-4CR reaction to obtain 23 new synthetic 2,5-diketopiperazines, which were tested against protozoan parasites of tropical diseases (Figure 4). For leishmaniasis (*L. infantum*), only two compounds were defined as partially active (compound **14**) and active (compound **15)**, showing good IC_50_ values among those tested but not surpassing the drug currently used (Amphotericin B). However, they showed high SI (>40). The authors did not identify a structure–activity relationship (SAR) for the substitution patterns on the aromatic rings of the 2,5-diketopiperazines synthesized. However, the presence of a hydrogen bond donor (HBD) in aldehyde phenyl rings seems to be important to obtain an active compound, since the compounds with 4-((methoxymethyl)­amino)­benzaldehyde or 4-hydroxybenzaldehyde lose the activity when replaced by 4-(dimethylamino)­benzaldehyde. This characteristic should be explored in future work, as should optimization of the scope using alkyl aldehydes. Furthermore, an expanded scope with greater variations of isocyanides and amines is suggested, seeking to establish a structure–activity relationship.

**Figure 4:**
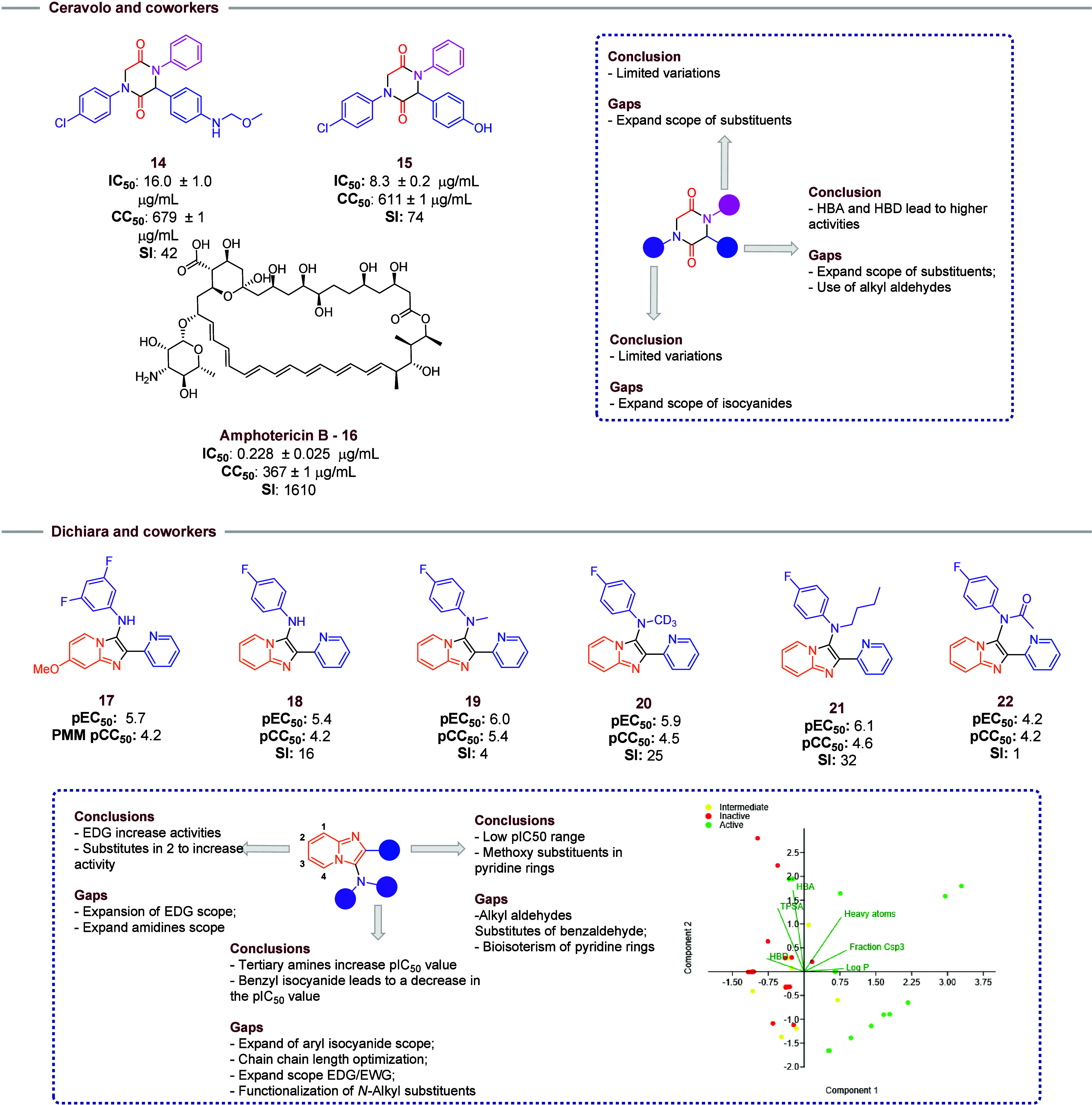
Selected compounds for Ceravolo and co-workers and Dichiara and co-workers. Ceravolo and co-workers highlight the principal active compounds and suggestions to new studies. Dichiara and co-workers highlight the principal findings and suggestions for new studies. Also, the reported PCA analysis conducted based on pEC_50_ values (Inactive: pEC_50_ *<* 5; Intermediate: 5.5 *<* pEC_50_ *<* 6; Active: pEC_50_ > 6), which indicates that compounds with a higher LogP and a lower flat characteristic should have a higher activity.

Dichiara and co-workers^12^ (Figure 4) used the same GBB methodology to optimize the results obtained in Akao and co-workers’ study^6^ using compound **17** as an initial hit to design new compounds against *L. infantum*. Other substituents in 2-pyridinecarboxaldehyde and other heterocycles were used. Their findings also show that the bioisosteric replacement of the pyridyl ring for imidazole, pyrazole, or pyridazine decreases the antileishmaniasis activity and results in a higher cytotoxicity. Nonetheless, the use of alkyl aldehydes or alkyl heterocycles still needs new studies. In imidazo­[1,2-*a*]­pyridine rings, a limited scope of EDG was tested, and it was identified that these substituents lead to a decrease in pEC_50_ values. In further works, the scope of EDG and EWG, the use of other amidines, and substituent positions in the ring should be increased. However, the N-alkylation process is a highlight (compounds **18**–**22**). The increase of lipophilic characteristics in this portion leads to a higher activity with good values of pCC_50_. An *N*-isobutyl chain leads to a compound with activity and SI higher than those of the initial hit **17**. So, in new studies, the influence of a more lipophilic alkyl isocyanides should be determined in activities or the functionalization of alkyl chains used in N-alkylation process. Using these pIC_50_ values reported by Dichiara and co-workers,^12^ and physicochemical characteristics calculated via SwissADME platform,^15^ a principal content analysis (PCA) was conducted and a clear separation of compounds with higher Csp^3^ characteristics and lipophilicity is observed. These findings suggest that the extension of studies of this type of modification is strongly recommended.

#### 2.1.2 Malaria

Malaria is a tropical disease caused by protozoa. Five species of Plasmodium can cause the disease, with *Plasmodium falciparum* (*P. falciparum*) being the most virulent. The WHO reports around 247 million cases of malaria worldwide, and although there are drugs available for treatment, plasmodium parasites have become more resistant over the years, making effective treatment more difficult.^14,16^ In Figure 5, two recent works with compounds obtained through Ugi reactions are highlighted.

**Figure 5:**
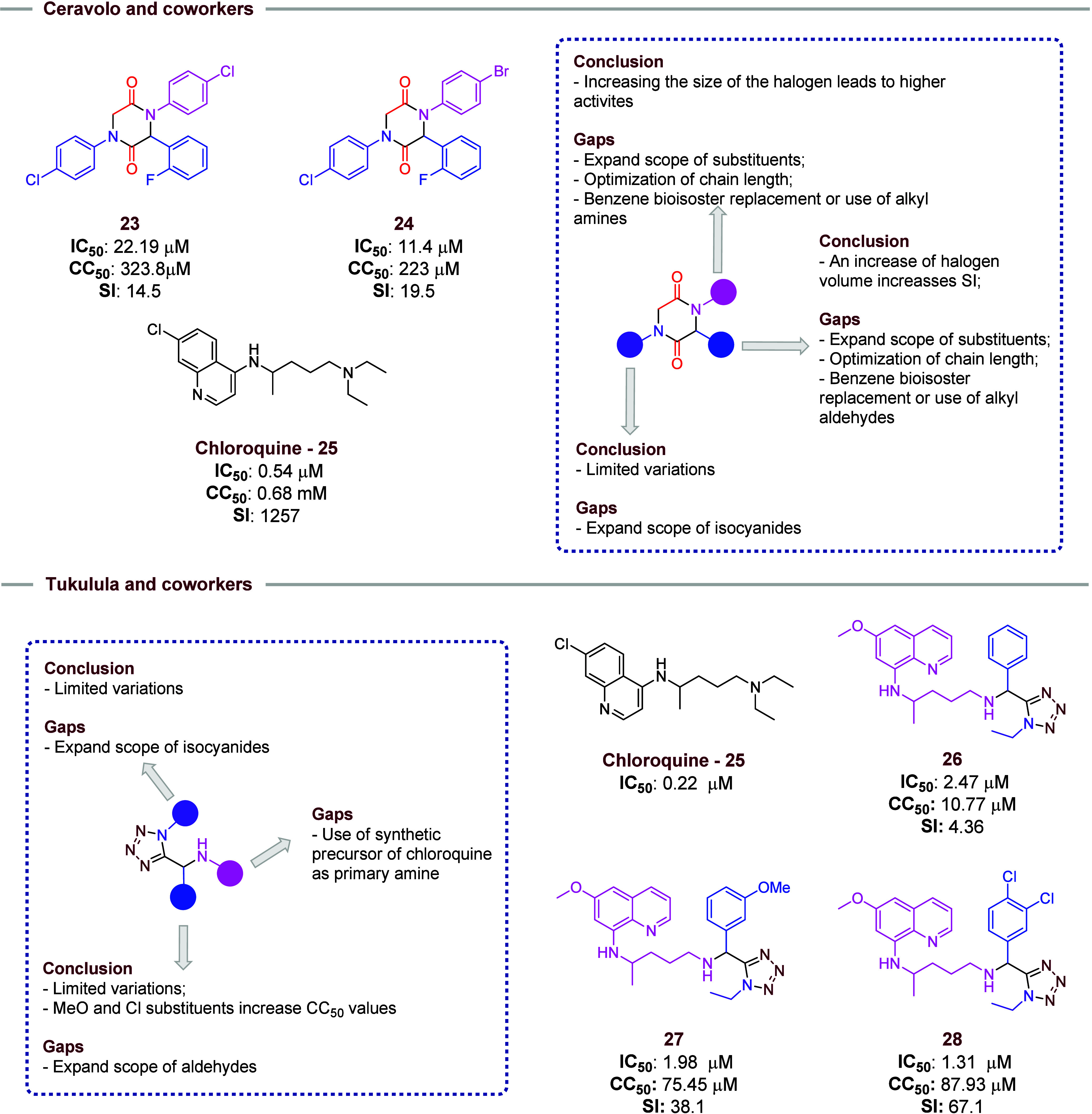
Selected compounds in the studies of Ceravolo and co-workers and Tukulula and co-workers. In both sections, the key findings and possibilities for further studies are highlighted.

Ceravolo and co-workers^14^ tested their synthetic 2,5-diketopiperazines for malaria (*P. falciparum*). Of these, compounds **23** and **24** showed the most potent antimalarial activity (Figure 5) with IC_50_ < 10 μM, low cytotoxicity, and a high SI. However, neither showed better results than chloroquine (**25**). In the evaluated 2,5-diketopiperazines, the activities increased as the size of the halogen substitutes was increased. This suggests that this is an important point to be evaluated in more detail in further studies. Also, an expanded scope of substitute, optimization of chain length, and benzene bioisosteric replacement should also be evaluated. *Para*-substituted benzaldehydes with halogen present an influence on the IC_50_ and CC_50_ values. With the increase of halogen size, the SI value increases as well. This profile characteristic should be more explored in further works, as well as an expanded scope of substituents, optimization of chain length, and bioisosteric replacement of the benzene ring. These suggestions should be explored as well in different isocyanides, since a few examples were evaluated.

Tukulula and co-workers^16^ synthesized a second generation of tetrazoles incorporating 8-aminoquinoline, using the Ugi-Azide reaction in their preparation (Figure 5). None of the five synthesized compounds showed superior antimalarial activity compared to that of chloroquine (**25**). The presence of methoxy (**27**) or chlorine (**28**, the most active compound with IC_50_: 1.31 μM) substituents in the benzaldehyde ring increases the CC_50_ and decreases IC_50_ values as compared to those without substituents (**26**). In further work, other examples of EDG and EWG should be evaluated in aldehydes. Given the reduced number of isocyanide examples used, it is not possible to define any influence, and this should be more detailed in the future. Also, the use of a chloroquine synthetic precursor should be used in the synthesis of new tetrazoles as the amine reagent in the Ugi-Azides reaction, aiming to increase activities. In addition to the tests for malaria, the authors also applied the same compounds to African trypanosomiasis (*T. brucie*), but none of the compounds showed better or similar results to the drugs used and no information regarding the structure–activity relationship was provided.

#### 2.1.3 Chagas Disease

Chagas disease is a neglected disease that affects Latin American countries. It is caused by the parasite *Trypanosoma cruzi*, and the main form of transmission is via the barber bug.^14^ As with the other diseases mentioned in this section, the drugs currently approved are linked to side effects and the resistance of these parasites to them.

Ceravolo and co-workers^14^ tested their synthetic 2,5-diketopiperazines derivatives, mentioned above, for Chagas disease (*T. cruzi*). Compounds **29** and **30** (Figure 6) showed antitrypanosomal activity with high selectivity values, although they did not have lower IC_50_ values when compared to benznidazole (**31**). Unfortunately, the results did not show a structure–activity relationship for these compounds when tested on *T. cruzi*, but some influence of aldehyde ring substituents is observed. The same halogen size–activity relationship observed in antimalaria activity is observed in this context. In addition, the presence of an HBD (hydroxyl group) increases the cytotoxicity of the compound when compared with the HBA analogue (methoxy group). A more detailed study with more examples of isocyanides and amines should be evaluated in further studies. In aldehydes, an expanded scope of substitutes should be evaluated as well.

**Figure 6:**
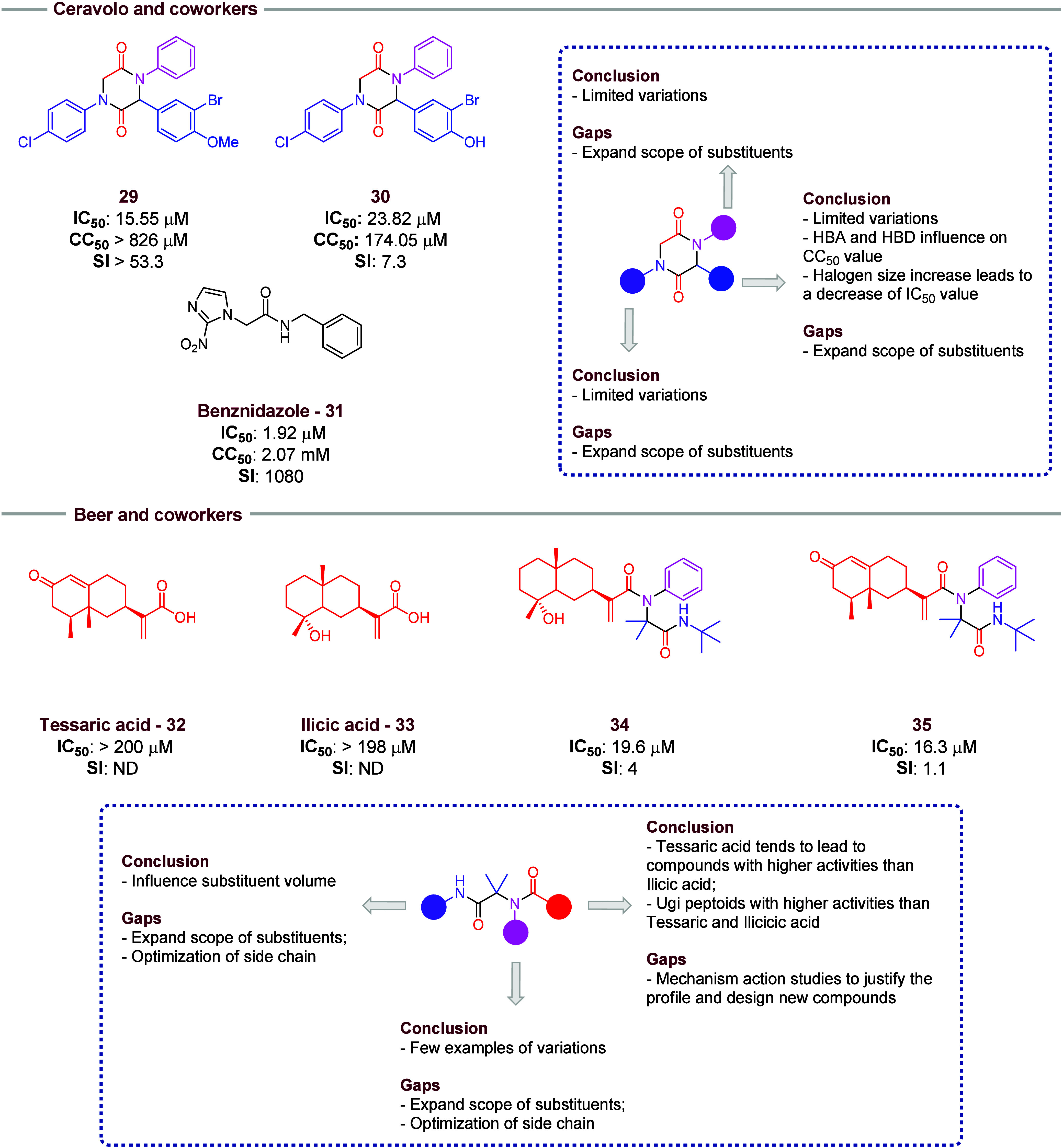
Highlights of compounds with anti-Chagas activity obtained by cyclization of Ugi adducts (**30** and **31**) in Ceravalo and co-workers’ publication. In Beer and co-workers research, tessaric acid derivatives (**34** and **35**) and ilicic acid derivatives (**26**) are highlighted.

Beer and co-workers reported a series of oxy-nitrogenated derivatives through Ugi reactions that were tested against *T. cruzi* epismatigotes^17^ (Figure 6). As the acid portion, the authors used tessaric and illicic acids (**32** and **33**), isolated from *Tessaria absinthioides* and *Flourensia oolepis*, respectively. All synthesized compounds showed higher activity than both natural acids. However, tessaric acid tends to lead to compounds with higher activities than ilicic acid (compounds **34** and **35**). However, the mechanism action of compounds is unknown, and this should be considered in future research to understand the higher activity of tessaric acid derived compounds. A few examples of amines were used in this work, and a more expanded scope should be considered in further work. Nevertheless, the activity of compounds decreases with more bulky groups in the isocyanide portion, which could be used as a starting point to guide new studies. These same compounds were also tested for antiproliferative activity in solid tumors, but they showed only moderate results.

### 2.2 Bactericide

New strains of bacteria resistant to classical antimicrobial drugs are a mandatory issue, and the optimization or synthesis of new compounds with potential activity has become the aim of some research groups. The compounds are typically tested using the ESKAPE panel, which contains a bacterial strain series with significant resistance to drugs that are commercially available.

Sapegin and co-workers^18^ (Figure 7) used a GBB methodology to obtain a new series of imidazo­[1,2-*a*]­pyridines and tested its activity against the ESKAPE panel. A set of 13 examples were synthesized using distinct substituted amidines. As an initial screening, the compounds had their activities tested by an inhibition zone (IZ) assay. Compounds with an appreciable IZ value were selected for minimum inhibitory concentration (MIC) assays. In many cases, the synthesized compounds showed similar or greater activities than controls such as furazidine (compound **39**), and the best results were observed against *Staphylococcus aureus.* In different strains, the presence of EDG substituents in 2-aminopyridine leads to a decrease in activity (compounds **36**–**38**). However, the use of other amidines, such as 2-aminopyrimidines or 2-aminothiazoles, is tolerated. In further investigations, the scope of EDG and EWG should be increased for a better understanding of influence. In the isocyanide portion, the influence of side chains is not possible to be defined, which is a clear point that needs further work to comprehend its influence. In this study, the authors used the 5-nitrofuran-2-carbaldehyde in all examples; in future research, it is recommended to use bioisosteric replacement of this heterocycle ring to better understand its influence.

**Figure 7:**
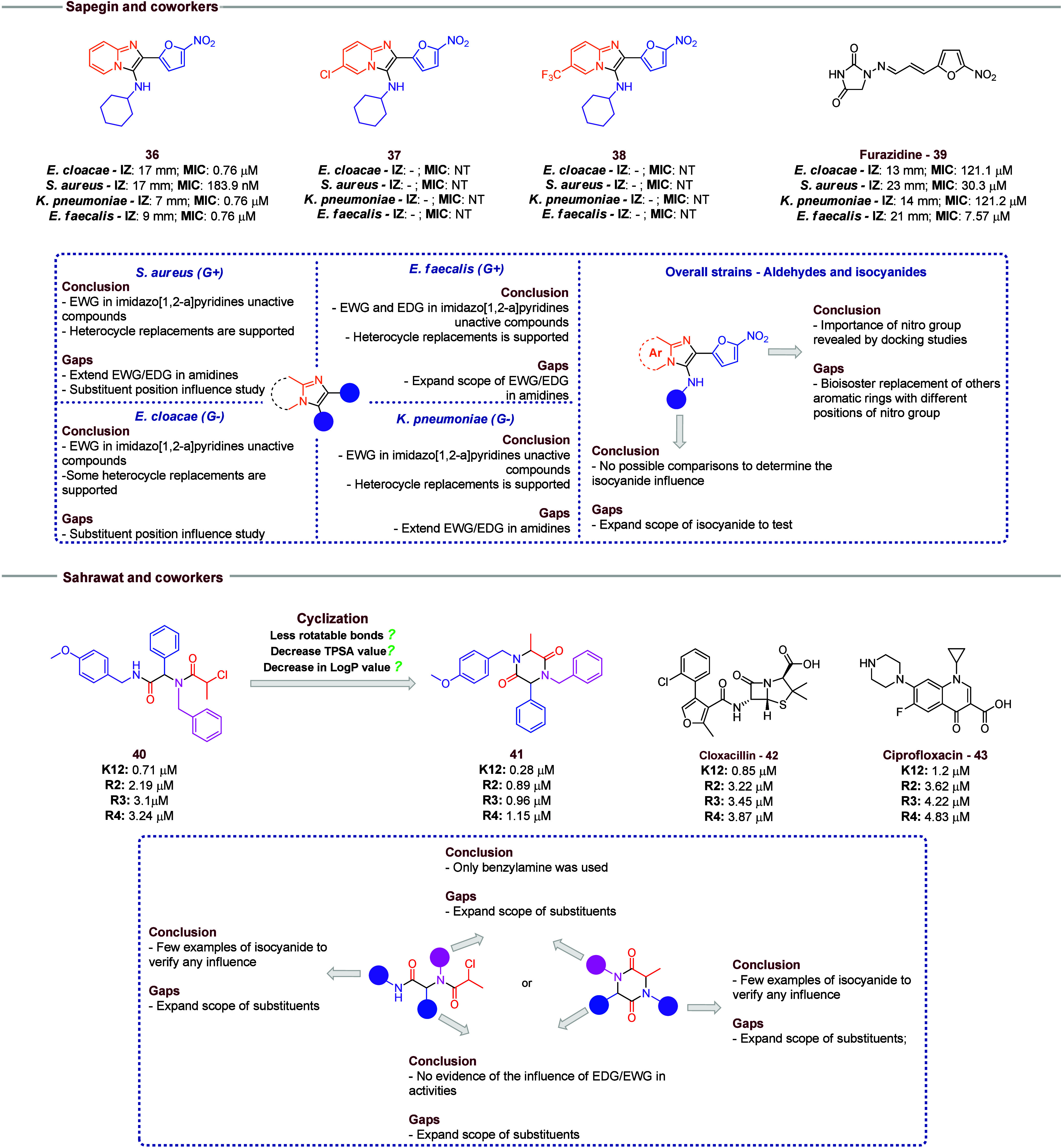
Selected compounds from Sapegin and co-workers and Sahrawat and co-workers. The influence of EDG and EWG against the ESKAPE panel is highlighted. Also, the possibilities of future research are emphasized. Sahrawat and co-workers’ publication demonstrates the higher potency of cyclic Ugi products when compared to the open chain derivatives (**40** and **41**) in different strains of *E. coli*.

Sahrawat and co-workers^19^ employed the Ugi reaction followed by a cyclization step to synthesize diketopiperazine derivatives with antibacterial activity against *Escherichia coli*, specifically targeting strains K12, R2, R3, and R4 (Figure 7). The cyclical derivatives presented greater activity than the uncyclized analogues (compounds **40** and **41**). Against R2–4 strains, cyclic compounds presented greater activities than cloxacillin and ciprofloxacin (**42** and **43**, respectively). Nevertheless, the study can be considered an initial point to design new compounds against these strains. This is justified by the lack of side chain diversity in the aldehydes, amines, and isocyanides used in this study.

### 2.3 Antivirals

#### 2.3.1 Covid-19

Since 2020, the world has applied significant efforts to develop tools to control the pandemic scenario, and vaccines were an important outcome of these initiatives. However, even after the critical pandemic stage, research on new compounds with anti-COVID-19 activity remains a mandatory issue, particularly regarding mutations and emerging SARS-CoV-2 variants with vaccinal escape.

The Main Protease (M^Pro^), a cysteine protease, was targeted due to its crucial role in the viral replication process.^20^ Additionally, this enzyme is conserved across various strains of SARS-CoV, and it is essential for the design of compounds with broad-spectrum activity. These reasons justify many efforts to design the inhibitors of it. Nirmatelvir (**43**), the first FDA drug approved against COVID-19, is a covalent inhibitor of M^Pro^. With a *K*_i_: 3.11 nM, the drug shows potent activity against the enzyme.^21^ However, the synthetic procedure to obtain Nirmatrelvir (Scheme 1) involves the use of structurally complex starting materials with a global yield of 49%. Also, Ritonavir (**44**) is coadministered with Nirmatrelvir because it plays the role of inhibitor of the CYP3A4 enzyme, a member of the cytochrome P450 superfamily enzyme, which increases the half-life of **43**. Nonetheless, the presence of **44** leads to possible co-interactions with other drugs, leading to a health risk to the patient.^21^ Because of it, the design of new covalent or noncovalent M^Pro^ inhibitors should be considered by the scientific community. In the examples below, many new inhibitors were obtained via the Ugi-4CR approach. In some cases, the same nanomolar-level activity was observed.^20,22^

**Scheme 1:**
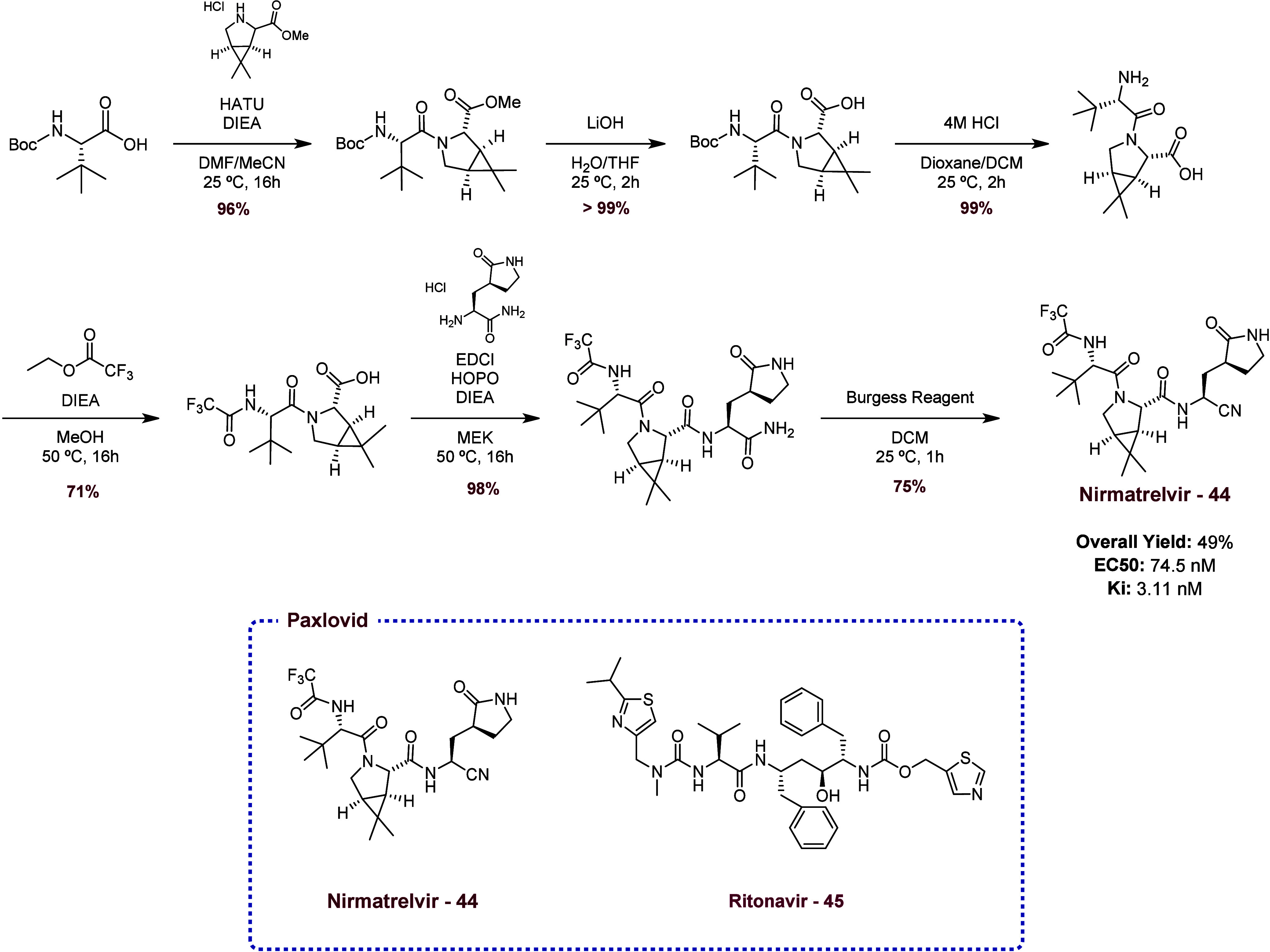
Synthetic Approach to Obtaining Nirmatrelvir, the Active Pharmaceutical Ingredient of Paxlovid[Fn sch1-fn1]

a
**HATU**: O-(7-azabenzotriazol-1-yl)-N,N,N′,N′-tetramethylu ronium hexafluorophosphate; **DIEA**: diisopropyl-ethylamine; **HOPO**: 2-hydroxypyridine 1-oxide; **MEK**: methyl ethyl ketone; **EDCl**: 1-[3-(dimethylamino)­propyl]-3-ethylcarbodiimide hydrochloride; **DMF**: N,N-dimethylformamide; **THF**: tetrahydrofuran; **MTBE**: methyl *tert*-butyl ether

Kitamura and co-workers (Figure 8)^23^ used a Ugi-4CR approach to design a novel noncovalent M^pro^ inhibitor. Compound **46**, previously reported by Jacobs and co-workers,^16^ served as a hit. Within 40 molecules, numerous modifications were evaluated in all of the molecule moieties. Among these, compound **47** showed the best results (IC_50_: 0.66 ± 0.07 μM; CC_50_ > 200 μM), and its diastereoisomeric mixture was purified via HPLC. Subsequent assays with the isolated compounds showed that the (*S*,*R*) isomer showed a higher affinity for the M^pro^, whereas the (*S*,*S*) isomer showed low affinity. X-ray analysis identified relevant interactions with Gly-143, Asn-142, and His-163. Moreover, the diastereoisomeric mixture was submitted in inhibition assays with different viral and human proteases. The synthesized hit displayed a high affinity exclusively for SARS-CoV and SARS-CoV-2 proteases among the tested systems. Regarding human proteases, the compound showed a consistent preference for M^pro^ interactions compared to the control.

**Figure 8:**
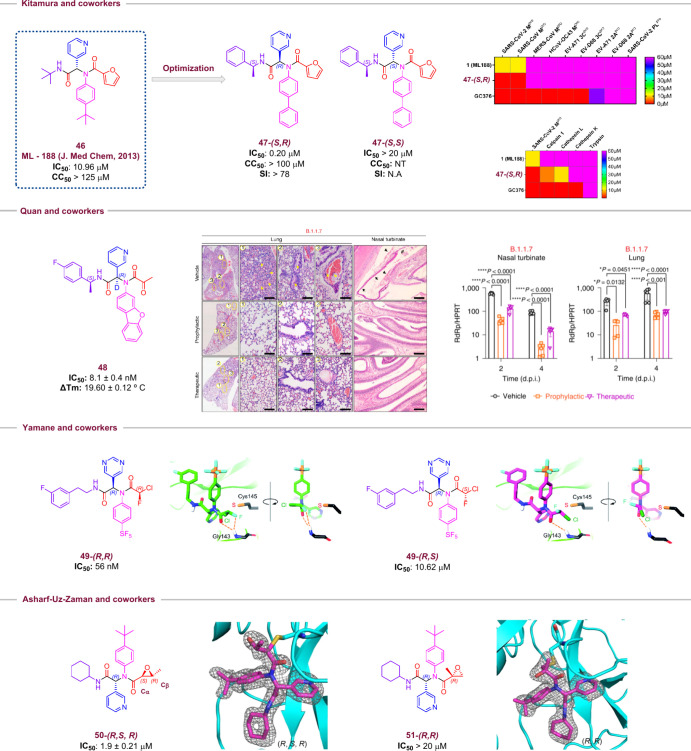
Kitamura and co-workers optimize the lead **46** to obtain a series of novel M^pro^ inhibitors. Compound **47** was the most active inhibitor synthesized. After a diastereoisomeric resolution, it was confirmed that **47-(*S*,*R*)** was the most active. In a series of inhibitions of viral and human protease assays, it was confirmed that compound **47-(*S*,*R*)** showed a higher selectivity for SARS-CoV’s proteases than human proteases. Compound **48** was the most potent covalent inhibitor obtained in Quan and co-workers’ study. Histological analysis demonstrated that **48** protects lungs and nasal turbinate tissues. Also, the therapeutic and prophylactic treatment showed a reduction in viral gene copies in the same tissues. Yaname and co-workers synthesized a novel series of covalent M^pro^ inhibitors. After diastereoisomeric resolution, compound **49-(*R*,*R*)** demonstrated the most active profile. Molecular docking studies justify these differences by the favorable position of **49** undergoing an attack of the Cys-145 residue. Ashraf-Uz-Zaman and co-workers investigate a broad scope of warheads to attack Cys-44 and Cys-145 residues. The best results were observed when epoxides were used. The Cys-145 demonstrated a preference to attack the Cα than Cβ; this is demonstrated by the lowest IC-50 values of compounds with Cα (**50-(*R*,*R*)**) substituted in comparison to Cβ (**50-(*S*,*S*)**); this was also confirmed in X-ray analysis.

Quan and co-workers (Figure 8)^20^ also employed Ugi-4CR to synthesize a new M^pro^ covalent inhibitor. Among the warheads evaluated, 2-oxopropanoic acid presented the best results. This portion was designed to act as an acceptor warhead for nucleophilic attack by Cys-145. Compound **48** presented the most promising results (IC_50_: 8.1 ± 0.4 nM; Δ*T*_m_: 19.60 ± 0.12 °C). The compound showed excellent bioavailability in mice (92.9%) and dogs (85.7%) and presented moderate bioavailability in rats (31.9%). Besides, it showed broad activity against different SARS-CoV-2 variants. According to *in vivo* mice assays, the compound significantly reduced viral proliferation in the nasal turbinate and lungs. Furthermore, immunohistochemistry confirmed the reduction of tissue damage in these regions.

Yamane and co-workers (Figure 8)^24^ used chlorofluoracetic acid in Ugi-4CR to obtain a series of covalent inhibitors. The first step involved the evaluation of the structure–activity relationship focusing on hydrophobic aniline *para*-substituents, with pentafluorosulfanyl (SF_5_) yielding good results in this screening. Various nitrogen-based six-membered heterocycles were evaluated as aldehydes as well, and 5-pyrimidine showed the best results. In addition, the isocyanide moiety showed that replacing the 3-fluorophenyl group with either a hydrophobic (*tert*-butyl) or bulky (7-isoquinoline) group reduced the potency, whereas the 3-pyridyl group was tolerated. Consequently, compound **49**-**(*R*,*R*)** exhibited the best results, and it was selected for chlorofluoracetic acid chirality evaluation. It was demonstrated that the *R* isomer shows a stronger affinity than that of the *S* form. Computational studies indicated that the *S* form of compound **49**-**(*R*,*S*)** is unfavorable to an Sn2 attack from Cys-145.

In the same way, to obtain a novel M^pro^ covalent inhibitor, Ashraf-Uz-Zaman and co-workers (Figure 8)^22^ designed compounds aiming to explore the nucleophilicity of another cysteine residue: Cys-44. Molecules containing phenyl rings with chloroacetamides or epoxide substituents exhibited low affinity for M^pro^, demonstrating the weak nucleophilicity of Cys-44 compared to Cys-145. However, compounds with an epoxide group derived from the carboxylic acid side chain showed significant M^pro^ affinity. A series of epoxides was evaluated, revealing higher affinity in epoxides without Cα substitutions (compound **50**). The steric hindrance at Cβ was tolerated since bulky substitutions at this position (compound **51**) did not prevent the opening of the epoxide by the nucleophilic attack of Cys-145 at Cα, as revealed by X-ray analysis.

Given these examples, it is clear that the use of Ugi-4CR approaches to design new M^Pro^ inhibitors is possible. In all three works that designed covalent inhibitors, the warhead is in the corresponding carboxylic acid side chain. In future work, a replacement of warheads should be investigated. Vankadara and co-workers conducted this study on the replacement of the nitrile warhead of Nirmaltrevir, and great results were observed, such as the increase of activity and a lower cytotoxicity.^25^ Also, the stability of the compounds against CYP3A4 and other relevant metabolic enzymes should be evaluated.

#### 2.3.2 Hepatitis C

The Hepatitis C Virus (HCV) infection is a liver illness that affects approximately 3% of the global population, with around 80% of cases tending to progress to chronic liver disease.^26^ Since 2011, treatment against HCV has been conducted through a combination of direct-acting antivirals (DAAs). Despite the various compounds developed and approved by the FDA for this purpose, challenges related to drug interactions and side effects persist, requiring further research to develop alternative compounds without these adverse effects.^26^

Han and co-workers^26^ developed a series of bisamide analogues (Figure 9), using the Ugi-4CR methodology, with anti-HCV activity by inhibiting cyclophilin A (CypA). For the SAR proposal, the authors introduced structural modifications based on the bisadine hit **51**, which had demonstrated anti-HCV activity in a previous study. In this approach, the indole and cyclohexyl portions were preserved, since they contributed to hydrophobic interaction and catalytic pocket filling in CypA, respectively. Structural modifications were restricted to the aldehyde portion, incorporating di- or trimethoxy groups. Among the synthesized analogues, five bisamides showed enhanced anti-HCV activity (EC_50_) and lower cytotoxicity (CC_50_). However, only compound **53** demonstrated a hydrophobic interaction with the gatekeeper pocket. Unlike compound **52**, the presence of a carbon chain between the phenyl ring and the nitrogen in the amine position caused the compound to invert into the adjacent gatekeeper pocket. The strength of the hydrogen bond between the indolylmethyl and His-54 of CypA led to increased affinity, while the electrostatic charge of the dimethoxyphenylethyl portion exhibited a favorable alignment into the gatekeeper site. Moreover, the trimethylphenyl moiety also established a hydrophobic interaction with the adjacent Ala-103, a crucial residue for the CypA pocket conformation. Given the singular binding pattern of compound **53** and the better EC_50_ and CC_50_ values in comparison to Ribavarin (**54**), a drug approved for use in Hepatitis’ treatment, it can be considered a promising candidate for anti-HCV therapy due to its potential as a CypA inhibitor.

**Figure 9:**
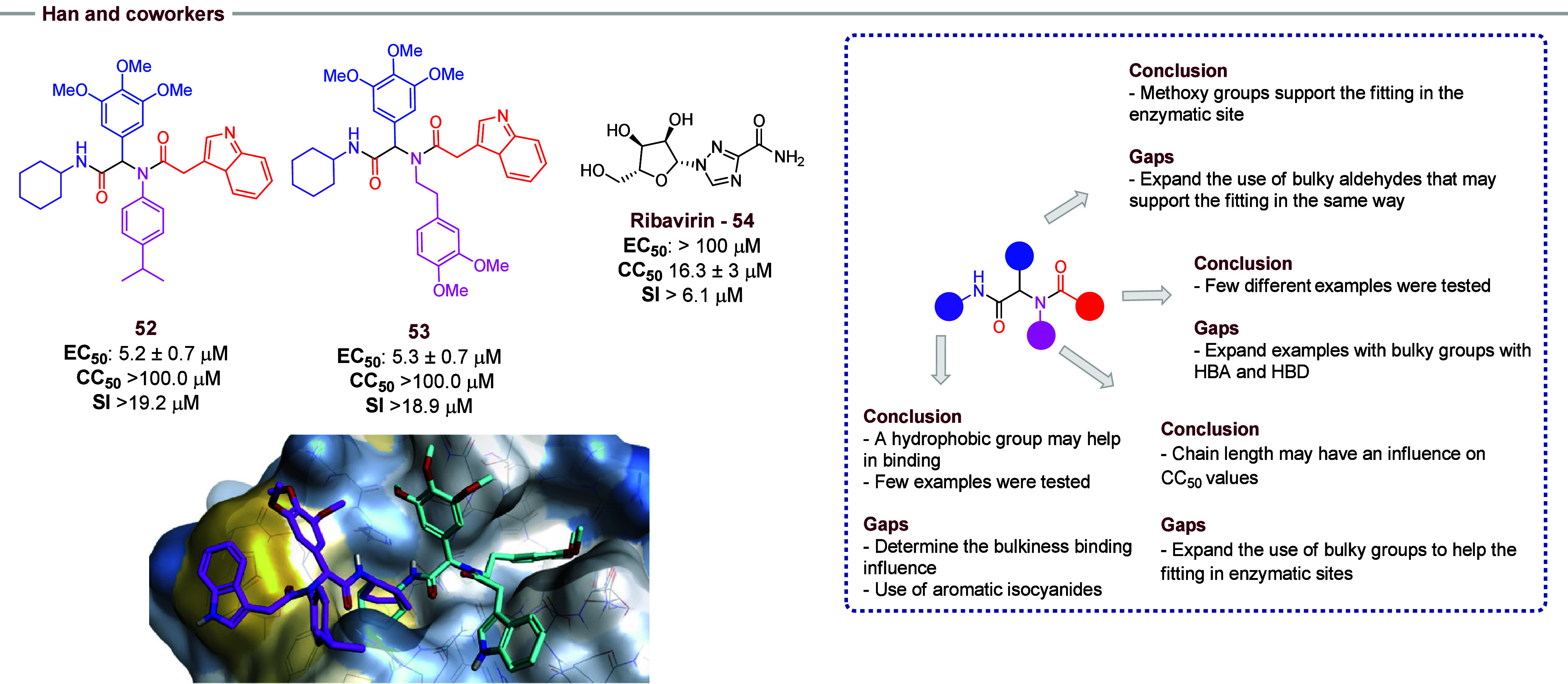
The highlight is compound **53** with antiviral activity against hepatitis C, obtained via optimization of the structure of compound **52**. On the side, an overlay of compound **52** (pink) and **53** (cyan) are associated with human CypA.

In further work, the use of aryl isocyanides should be considered. Also, the influence of bulky groups in binding should be evaluated in new examples of isocyanides and aldehydes. Aiming for a bioisosteric replacement of the indole ring, other large groups with HBA and HBD could increase the activity of compounds. In the amine moiety, the chain length may have an influence on CC_50_ values, and by this, the use of other bulky groups may help the fitting in the enzymatic site, which should be evaluated in new research.

#### 2.3.3 HIV

The human immunodeficiency virus (HIV) attacks the body’s immune system through white blood cells (CD4). Despite advances in treatment, HIV remains a significant global public health issue, with around 630,000 deaths due to complications of the disease in 2022 alone. Although there is no cure, HIV can be managed using antiretroviral therapy, allowing those affected to maintain a tolerable to good quality of life.^27^

Ji and co-workers^28^ used compound **55** as a prototype and synthesized 18 new phenylalanine-containing peptidomimetic molecules employing the Ugi-4CR reaction. The entire set was tested against HIV-1 and HIV-2. Some compounds (**57–60**, Figure 10) showed antiviral activity against HIV-1 in single-digit micromolar concentration, but none of the compounds **57–60** emerged as active as **55** and nevirapine (**56**), both used as controls, even with great EC_50_ values in some cases. Regarding activity against HIV-2, only compounds **59** and **60** showed results similar to compound **55**. Compound **60** presents a higher SI to HIV-2 than both controls, which is important as an increase in activity. The study showed that the substituents have a direct relationship with the antiviral activity and selectivity of the compounds. For instance, compounds containing the hydrophobic naphthalene ring, derivatives from carboxylic acid, in their structure showed good selectivity for HIV-1 and HIV-2. In further work, the influence of other fused rings should be considered. Also, some docking studies should be conducted to evaluate other possibilities of bulky HBA and HBD. Furthermore, the use of aminocyclopropane increases the CC_50_ values, and 4-cyanobenzylamines increases the HIV-2 selectivity, which could be an interesting focal point to design new selective HIV-2 inhibitors. In further work, the use of bioisosteres of the cyano group or the increase of π-orbital characteristics in this portion, using biaryl compounds, should be evaluated. In the same way, halogens may be responsible for obtaining selective anti-HIV-1 inhibitors. However, the evaluation of halogen substitutes should be considered to verify the influence of halogen size substitutes.

**Figure 10:**
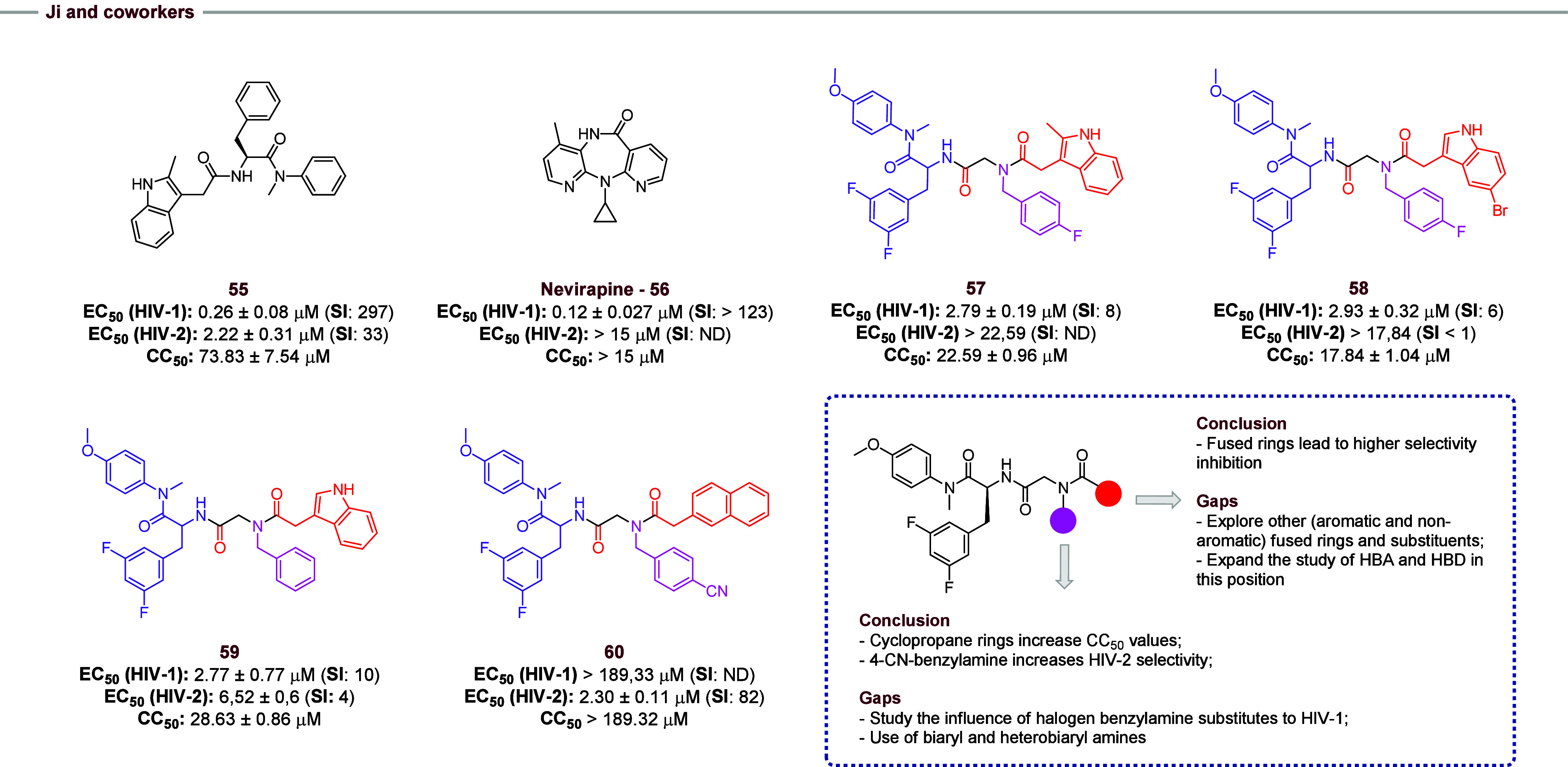
Compounds **57–60** were obtained by the Ugi-4CR reaction, using compound **55** as a prototype for optimization, and the respective antiviral activity against HIV-1 and HIV-2 was obtained in the work of Ji and co-workers.

## 3 Anticancer

Cancer accounts for 16.8% of deaths and 22.8% of noncommunicable diseases worldwide. These numbers reinforce the growing interest in developing novel compounds with activity against a broad spectrum of cancers.^29^ For these reasons, the following presents some works that illustrate how multicomponent reactions can be used to obtain new compounds with a reduced number of synthetic procedures, with great values of anticancer activity.

Xu and co-workers used the Ugi-4CR reaction as a key step to obtain compounds designed to promote ferroptosis by increasing the lipid peroxides in tumor cells leading to glutathione peroxidase 4 (GPX4) inhibition.^30^ The study employed the scaffold hopping strategy to investigate structural modifications on the lead ferroptosis regulator compound **61**.^31^ The SAR of the synthesized compounds was determined, and molecular modifications involved ring opening and bioisosteric replacements (Figure 11).^30^ All prepared analogues were submitted to *in vitro* assays, and micromolar IC_50_ values were obtained. In the same direction, the GPX-4 inhibition potential of each compound was evaluated and the same micromolar scale was observed. Additionally, compound **62**, which showed the best results during the evaluation, induced an intracellular increase in lipid peroxide concentration, promoting a ferroptosis process. Supported by these results, *in vivo* experiments were conducted, and it was observed that tumor growth was reduced in groups administered 15 and 30 mg/kg of compound **62**. Compound **61** was used as a control during the work, and **62** showed better results than the control. Moreover, no cytotoxic effects were detected in the heart, liver, spleen, lung, or kidney tissues.

**Figure 11:**
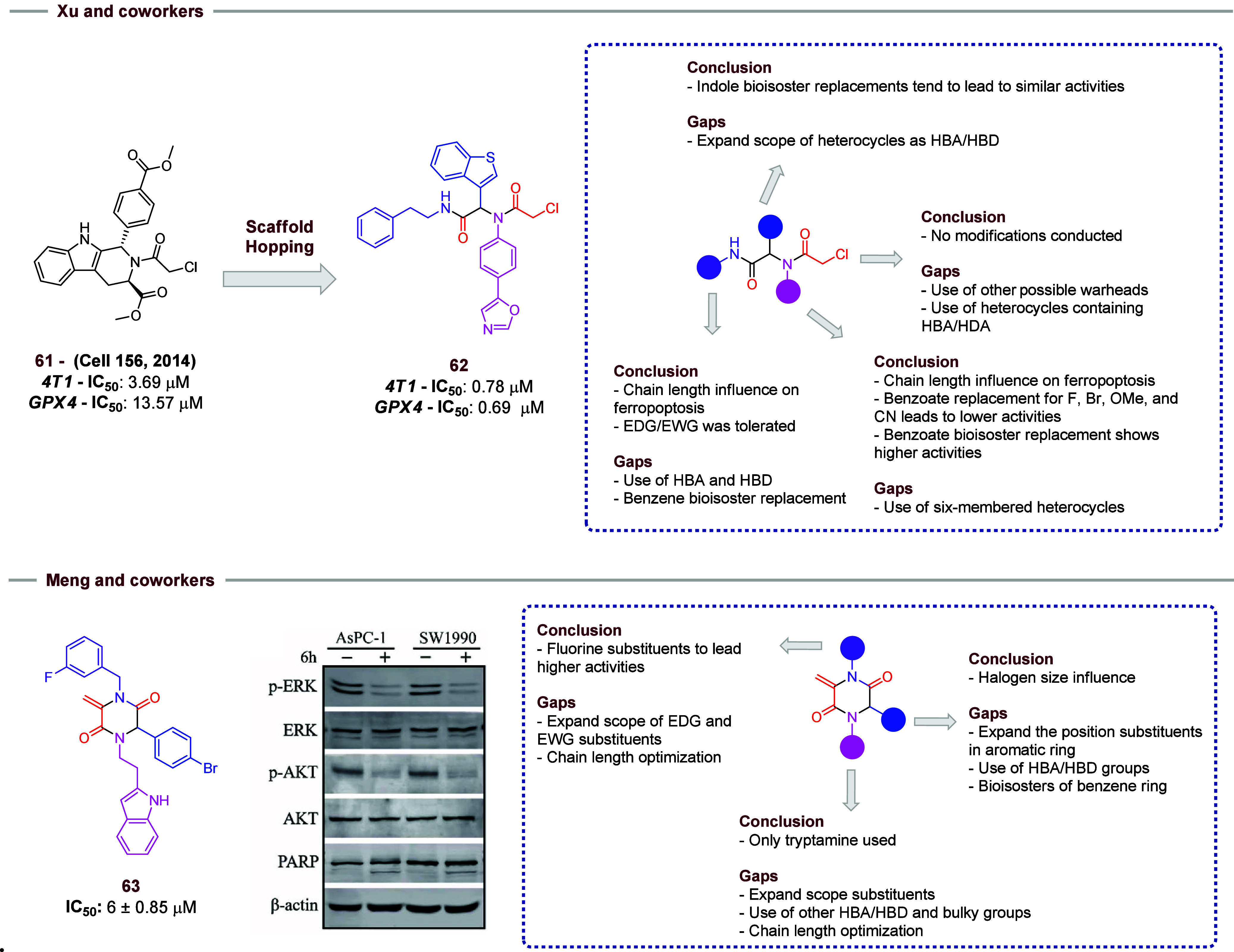
Compound **62** was obtained by Xu and co-workers using the Ugi-4CR reaction. New suitable modifications for new studies are highlighted in the square. Meng and co-workers obtained a novel series of tryptamine-piperazine-2,5-diones derivatives. Compound **63** was the most active compound against the AsPC-1 and SW1990 cancer cell lines. The Western blot assays detect the presence of proteins of apoptosis process and new suitable modifications for further studies.

In a SAR context, the chain length of isocyanides impacts ferroapoptosis, and the presence of EDG and EWG was tolerated. However, the use of HBA/HBD, as well as the benzene bioisosteric replacement, should be evaluated in further studies. The strategy of bioisosteric replacement was used to evaluate the influence of distinct heterocycles present in aldehyde starting materials, and it leads to great results. Nevertheless, the examples of heterocycles as well as HBA/HBD should be investigated in further research. In the amine side chain, it was detected that a chain length influences ferroapoptosis. The benzoate replacement for F, Br, MeO, and CN groups leads to reduced activity, but the replacement for benzoate bioisosteres leads to higher activities. The use of other biarylamines with a broader scope of substituents should be evaluated in further studies. In the carboxylic acid portion, only 2-chloroacetic acid was used in the presence of this portion in initial hit **61**. However, modifications in this portion are suitable for further studies. Use of other warheads or the use of heterocycles containing HBA/HBD groups are possible modifications in this moiety.

Meng and co-workers synthesized novel compounds with activity against pancreatic cancer cell lines^32^ (Figure 11). The tryptamine-piperazine-2,5-diones were obtained via a postcyclization Ugi-4CR. Among the 11 compounds synthesized, three of them showed higher activity against the AsPC-1 and SW1990 cell lines. Different benzaldehydes were evaluated. Notably, the size of the halogen substituent in the *para* position of aldehyde aromatic ring influenced the anticancer activity: larger halogens corresponded to higher activities (F < Cl < Br). Bromine-containing compound **63** showed a micromolar IC_50_ value of (6 ± 0.8 μM), and this suggested that **63** may induce apoptosis caused by the increased expression of proteins that are involved in this mechanism. In further investigations, a broader scope of aldehydes should be considered to verify the influence of them in activities. Use of distinct HBA and HBD groups in *para* positions may lead to modifications in IC_50_ values. Furthermore, the presence of fluorine substituents in benzyl isocyanide leads to higher activity. In future work, the same size–activity relationship should be evaluated. The expansion of the scope of EDG and EWG in aryl isocyanides should be explored in detail in further work. In the same way, the evaluation of a broader scope of amines should be considered, but in this work, only tryptamine was used.

Wang and co-workers used gossypol in GBB reactions to obtain a novel series of compounds with anticancer activity against a broad spectrum of cellular lines in a phenotypic assay (Figure 12).^33^ First, the influence of the isocyanide side chain was evaluated using 2-aminopyridine as amidine in the reaction and gossypol as aldehyde. It was found that lipophilic substituents lead to more active compounds. Bulky groups, such as adamantyl, yielded the highest IC_50_ values. In the ACHN cell line, the compounds tend to be inactive in the presence of this substituent. However, in the absence of a side chain in this portion, with a free NH_2_ in this moiety, behavior similar to that obtained in adamantyl was observed. The same inactive profile is observed in U251 cell lines. The best IC_50_ values, in this first optimization process, were observed when cyclohexyl isocyanide was used (compound **64**). However, the chain length optimization process and a broader scope of substituents in aryl isocyanides or the use of biaryl isocyanides were not evaluated and should be considered in further work. Subsequently, evaluation of 2-amipyridine substitutes was performed. At this point, a bioisosteric feature was observed: the presence of halogen atoms such as Cl and Br led to higher mean IC_50_ values when compared with a methyl group. The presence of a methyl group showed better results than the CF_3_ group or the absence of substituents. Also, the presence of a hydroxyl group led to an inactivated compound; however, no other HBD/HBA examples were evaluated. So, future works are encouraged to evaluate the influence in activity using a broader scope of HBD/HBA (or other examples of amidines) substituents as well other examples of EDG. Furthermore, the compounds were tested against the Bcl-2 protein family, which are regulators of apoptosis. It was shown that these compounds act as inhibitors of this class of enzymes, and molecular docking studies showed significant interactions of ligand–enzyme.

**Figure 12:**
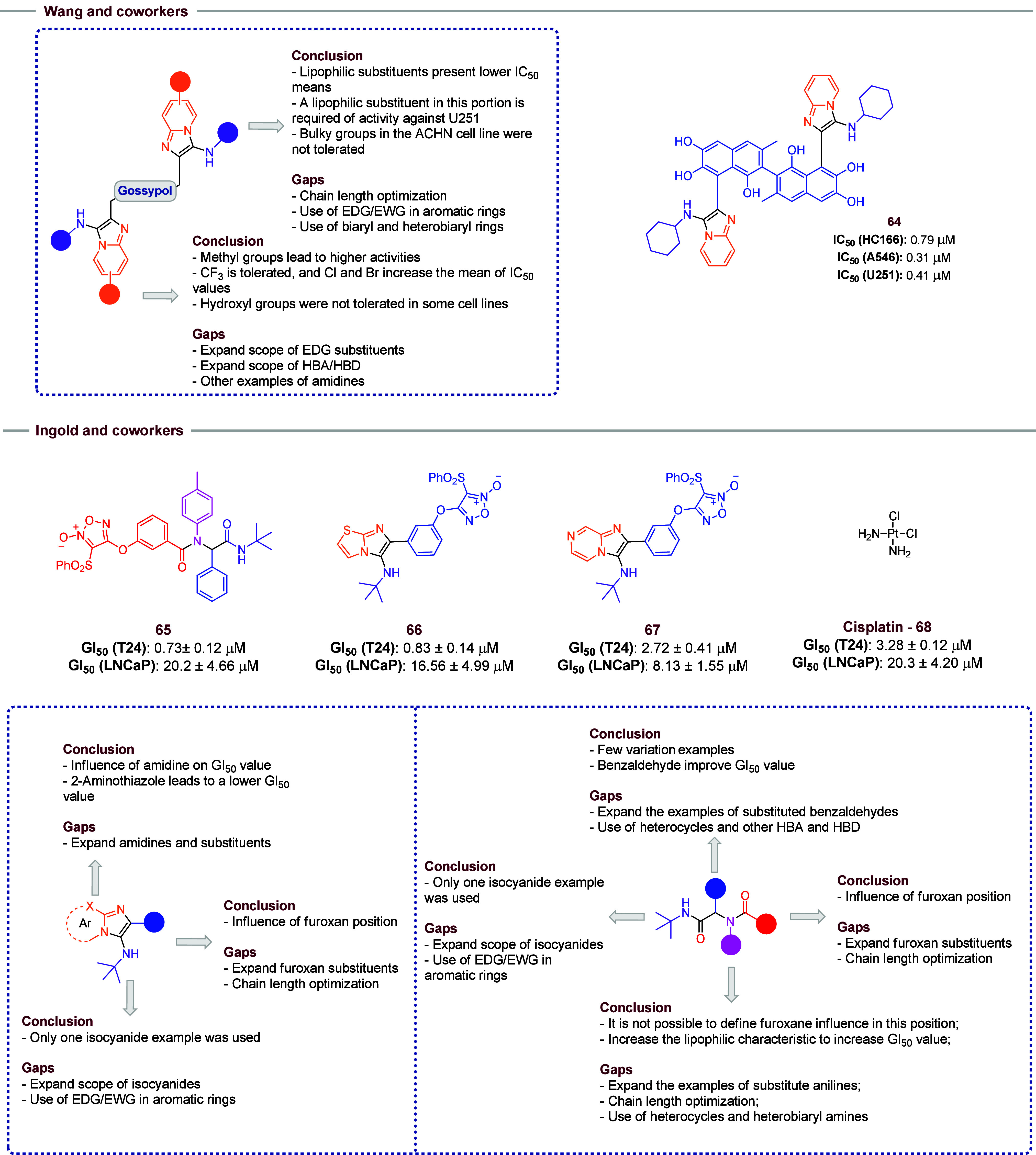
Wang and co-workers obtained compound **64** via GBB reactions where it presented the best activity value. The box with highlighted findings has some possibilities for further work. Ingold and co-workers obtained compound **65** via Ugi-4CR and **66**–**67** via GBB alongside them and presented the main conclusions of the work and some possible new features to be explored.

Ingold and co-workers^34^ synthesized novel compounds with activity against prostate cancer (LNCaP) and bladder cancer (T24) cell lines (Figure 12). The compounds were obtained via Ugi-4CR and GBB reactions. In Ugi products, all compounds featured a furoxan moiety in their carboxylic acid or aldehyde moieties, which is responsible for nitric oxide (NO) release in the medium, promoting cell cancer death. In GBB derivatives, furoxan is a substituent in the benzaldehyde ring. The 12 synthesized compounds demonstrated anticancer activity against both T24 and LNCaP cell lines and released NO in their presence as well (compounds **65**–**67**). The compounds showed better results than cisplatin (**68**) used as a control. The most significant results were, when compared with **68**, the reduction of cytotoxicity and the maintenance of the activity or decrease of GI_50_ values, leading to an increase in SI. However, different structural modifications in the GBB and Ugi reactions did not lead to significant changes in compound activity. A broad scope of substituents in reagents was not used; for this reason, a reduced number of influences can be identified. For the Ugi compounds, the position of furoxan substituents impacts on activity, and a *meta*-substituent (**65**) showed the best results. Other substituents in furoxans should be considered in further studies to evaluate how it can interfere in anticancer proprieties. For the GBB series, the presence of an ortho-furoxane group resulted in greater activity. In addition, the use of 2-aminothiazoles leads to a submicromolar activity compound (**66**) when compared to 2-aminopyridines derivatives (**67**). So, the exploration of a broader scope of amides should be considered in further works. Compounds lacking the furoxan moiety showed no activity. The study indicated that anticancer activity is not solely associated with the release of NO in all of the molecules studied, suggesting the involvement of additional mechanisms.

## 4 Future Outlook

Multicomponent reactions (MCRs) are a pivotal tool in the rational synthesis of new compounds and the optimization of synthetic procedures to obtain commercial drugs. Drugs approved by the FDA in the last 5 years indicate a clear commercial interest in oncology and infectious disease areas. In this context, Ugi and GBB reactions can serve as powerful tools for the design of new compounds.

As demonstrated in the COVID-19 section, the long synthetic procedure to obtain Nirmatrelvir, an active pharmaceutical ingredient of Paxlovid, leads to high production costs. Some compounds designed via Ugi reactions obtained similar activity profiles but with fewer synthetic procedures. This is a very significant advantage in the process of the optimization of costs on an industrial scale. Beyond that, the compounds tend to show a lower cytotoxicity profile, which is an important characteristic for a molecule that would be administered on a global scale.

In the parasitic infections section, it was demonstrated that compounds obtained via GBB reactions and evaluated for their antileishmanial profile showed higher activity than the controls. Beyond that, due to the high number of examples synthesized, it was possible to determine a more accurate influence of each portion of the molecule. Also, a principal component analysis was conducted, and a clear profile of physicochemical properties is relevant to compounds’ activities. This chemometrics approach could be analyzed in more detail in further studies. Quantitative structure–activity relationship (QSAR) studies should be conducted using the works summarized herein.

In the anticancer section, compounds obtained via these MCRs have shown promising results from *in vitro* to *in vivo* assays. Among the works, the contributions by Xu and co-workers is highlighted. The Ugi four-component reactions were used to obtain a compound with high activity against cancer via the ferropoptosis mechanism. Also, it was demonstrated that the administration of this compound *in vivo* studies reduces tumor weight and does not cause damage in relevant organs. This demonstrates that applications of the MCR approach could be applied to recently discovered mechanisms of actions.

The design and synthesis of new biologically active compounds will always be a mandatory issue to increase life expectancy, aligning with the United Nations’ Sustainable Development Goal (SDG) of “Good Health and Well-Being.” Thus, the demand for new compounds can be addressed through rational design, employing multicomponent reactions as a crucial synthetic step. Moreover, from both synthetic and industrial standpoints, this approach provides higher atom economy, cost reduction, and shorter timeframes for obtaining new bioactive compounds, thus contributing to the SDG of “Responsible Consumption and Production”.

For these reasons, Ugi and GBB reactions are likely to attract increasing attention due to their potential in addressing health challenges and contributing to sustainable development.
